# Effect of Berry Extracts and Bioactive Compounds on Fulvestrant (ICI 182,780) Sensitive and Resistant Cell Lines

**DOI:** 10.1155/2012/147828

**Published:** 2012-12-31

**Authors:** Denzel R. Woode, Harini S. Aiyer, Nicole Sie, Alan L. Zwart, Liya Li, Navindra P. Seeram, Robert Clarke

**Affiliations:** ^1^Lombardi Comprehensive Cancer Center, Georgetown University, Washington, DC 20057, USA; ^2^Columbia University, 5992 Lerner Hall, 2920 Broadway, New York, NY 10027, USA; ^3^Royal College of Surgeons in Ireland, Dublin, Ireland; ^4^Department of Biomedical and Pharmaceutical Sciences, College of Pharmacy, University of Rhode Island, 7 Greenhouse Road, Kingston, RI 02881, USA

## Abstract

Fulvestrant (ICI 182,780; ICI) is approved for the treatment of advanced metastatic breast cancer that is unresponsive to other endocrine therapies. Berries are frequently consumed for their antioxidant, anti-inflammatory, and anticancer potential. In this study, we tested the efficacy of two berry extracts (Jamun-EJAE and red raspberry-RRE) and their bioactive compounds (Delphinidin-Del and Ellagic acid-EA) to inhibit cell proliferation with or without a sublethal dose of ICI in various breast cancer cell lines. ICI-sensitive (LCC1, ZR75-1, and BT474) and -resistant (LCC9, ZR75-1R) cells were subjected to treatment with berry extracts alone (0.1–100 **μ**g/mL) or with a sub-lethal dose of ICI (<IC_50_ dose; 1 nM for sensitive; 1 **μ**M for resistant cells). Extracts and Del enhanced the effect of ICI in sensitive ZR75-1 and BT474 cells primarily in an additive fashion (measured by relative index (RI)~1). In ZR75-1R cells, both EJAE and RRE synergistically enhanced the effects of ICI (15–50%; *P* < 0.05; RI > 1). EA, in doses tested, did not have any significant effects on any of the cell lines. Finally, we found that the extracts were more effective at lower, physiologically relevant concentrations than at higher experimental doses.

## 1. Introduction

It is estimated that 28% of new cancer cases are breast cancer incidences [1]. Of these newly diagnosed breast tumors, 65–70% will express the estrogen receptor alpha (ER*α*) [2]. Estrogen activation of the ER is required in the development of a healthy mammary gland. However, it also can be involved in the development of both primary and secondary breast cancers due to altered ER signaling [3, 4]. Activation of ER by estrogen (E) promotes cell growth and survival of tumor cells [5]. Primary ER*α*-positive breast cancer can be effectively treated with antiestrogens (AE). AE drugs can be used in the metastatic, adjuvant, and chemopreventive settings and responses are typically seen in about 70% of ER+ patients selected for such treatment [4, 6]. Currently, Tamoxifen (TAM) is the most widely used AE for ER+ breast cancer. TAM is an example of a selective estrogen receptor modulator (SERM) which acts as an antagonist to ER*α* in the breast, leading to a reduction in the proliferation of tumor cells [6]. However approximately 1/3 of tumors treated with TAM either possess *de novo* or acquire resistance to TAM, leading to breast cancer recurrence [7]. Further, TAM acts as an ER agonist in the endometrium and in certain cases in the breast epithelium [4, 8, 9].

Fulvestrant (Faslodex, ICI 182, 780; ICI) is a steroidal AE designed to have no agonist activity with the ER [10]. ICI acts by degrading, and downregulating the ER*α* in the tumor cells [10, 11]. Currently, ICI is used for the treatment of advanced breast cancer that is resistant to other endocrine therapies. It is effective in tumors and cell lines that are resistant to TAM yet still express ER [12]. However, in the clinic the duration of response (DoR) and time to progression (TTP) on ICI is only 19 and 5.5 months, respectively [13]. Finding strategies to increase the sensitivity of the breast cancer cells to ICI may result in increased efficacy of drug therapy. 

There is evidence to show that healthy changes in diet can prevent up to 40% of breast cancers [14]. Further, data is beginning to show that an increased intake of fruits and vegetables in patients recently diagnosed with breast cancer may reduce the risk of recurrent breast cancer [15–17]. This preventive effect can largely be attributed to the various phytochemicals present in fruits and vegetables. These bioactive compounds have been shown to affect the development of both primary and secondary breast cancer by affecting cell proliferation, survival, and death [3, 18].

 Red raspberry (RRB) is a readily available fruit that is part of our diets and is a rich source of phytochemicals. It is composed of compounds that inhibit the proliferation of many types of cancer cells, including breast cancer [19–21]. RRB contains high levels of anthocyanins such as cyanidin-3-sophoroside, cyanidin-3-glucoside, pelargonidin-3-glucoside, and ellagic acid [3, 22]. *In vivo* studies in mice show that red raspberry diet increases DNA repair enzymes and reduces oxidative DNA damage. Ellagic acid, a phenolic component in RRB, also shows similar effects [23]. Further, the polyphenols present in RRB can inhibit nuclear receptors, growth factors, and kinase signaling leading to cell-cycle arrest, apoptosis, or autophagy-associated cell death [3]. The red raspberry extract (RRE) used in the study has been previously standardized and inhibits the growth of several cancer cell lines, including breast cancer, in a dose-dependent manner [22].

The Java Plum, also called the Jamun fruit, is the fruit of *Eugenia jambolana Lam*., a tree that can be found in Florida and Hawaii in the United States and other various tropical zones in the world. The Jamun fruit extract (EJAE) used in this study has been previously standardized [24]. EJAE is rich in anthocyanidins including petunidin, malvidin, delphinidin, cyanidin, and peonidin [24, 25]. EJAE reduces the proliferation of MCF7-aro (aromatase and ER*α* positive) and MDA-MB-231 (ER*α* negative) breast cancer cell lines [24]. Neither EJAE nor RRE has been previously tested in ER+, ICI-resistant cell lines. 

Clarke and coworkers have developed a series of cell lines as an *in vitro* model of AE resistance [6]. These cell lines have been used extensively to study the mechanisms of AE resistance. The LCC series were initially derived from MCF7 cells and consists of LCC1 (E independent, E stimulated and TAM and ICI sensitive) and LCC9 (E independent, E stimulated and TAM and ICI resistant) [26, 27]. More recently we have derived another ICI-resistant variant of the ZR75-1 cells (ZR75-1R), that was developed by culturing ZR75-1 in sequentially increasing concentrations of ICI for more than one year (A. Zwart and R Clarke, unpublished data). These cell lines serve as *in vitro *models that represent some phenotypes of ICI-resistant breast cancer.

In this study, we tested the effects of Jamun (EJAE) and red raspberry (RRE) extracts and the individual phenolics ellagic acid (EA) and delphinidin (Del) on ICI-sensitive (LCC1, ZR75-1, BT474) and resistant (LCC9, ZR75-1R) cells. We hypothesized that in sensitive cells, berry extracts and their compounds would have a synergistic effect with ICI, increasing the inhibition of cell proliferation by a sublethal dose of ICI (<IC_50_), and in resistant cells, the extracts would resensitize the cells to ICI making them more susceptible to ICI-induced growth inhibition. Cell proliferation was measured in the presence of extracts/compounds with or without ICI. In addition, molecular markers of apoptosis, autophagy, and ER signaling were also analyzed to understand the mechanism by which EJAE, RRE, and their compounds reduced the growth of these cells. 

## 2. Materials and Methods

### 2.1. Cell Culture

The LCC1 and LCC9 cells were cultured in modified IMEM (without phenol-red) containing 5% charcoal-stripped calf serum (E level < 10^−12^ M.) The ZR75-1, ZR75-1R, and BT474 cells were cultured in modified IMEM containing 5% fetal bovine serum (E levels ~10^−8^ M.) Establishment of LCC1 cells has been previously described [27]. LCC9 and ZR75-1R cells were derived by the long-term selection of LCC1 and ZR75-1 cells, respectively, in sequentially increasing concentrations of ICI as described [26]. All cells were grown in a humidified incubator at 37°C with 5% CO_2_. Cells were then harvested at 70% confluence for use in cell proliferation and biomarker analysis. All cell lines tested negative for *Mycoplasma sp*. contamination. 

### 2.2. Berry Extracts and Compounds

Berry extracts were obtained from Dr. Navindra P. Seeram (University of Rhode Island, RI) and have been previously described [24]. Briefly, EJAE was obtained by sequential extraction of freeze-dried Jamun fruit in cold hexanes, ethyl acetate, and acidified methanol [24]. It contains 3.6% anthocyanins measured as cyanidin-3-glucoside equivalents. EJAE is the same acidified methanol extract referred to as the Jamun fruit extract (JFE), in a previous publication [24]. RRE was extracted from red raspberry using acidified methanol as described [22]. Ellagic acid was purchased from Sigma Chemicals (St. Louis, MO) and delphinidin from Chromadex Inc. (Irvine, CA.)

### 2.3. Cell Proliferation Assay

 Cells were plated in 48-well plates (5,000 cells for LCC1 and LCC9; 10,000 for ZR75-1, ZR75-1R, and BT474) and incubated overnight for attachment. RRE, EJAE, EA, and Del were dissolved in DMSO and ICI was dissolved in ethanol. DMSO (0.1%) and ethanol (0.01%) were used as appropriate vehicle controls either alone or in combination. All cells were treated with 1–100 *μ*g/mL RRE, 0.1–100 *μ*g/mL EJAE, or 0.1–10 *μ*M EA or Del with or without ICI (1 nM and 1 *μ*M). For sensitive cell lines (LCC1, ZR75-1, and BT474), cells were treated with 1 nm ICI, which is a sublethal dose and well below the IC_50_ of ICI for LCC1. For resistant cell lines (LCC9 and ZR75-1R), ICI was used at a concentration of 1 nM and 1 *μ*M. All groups were treated after 24 h for the first time and the treatment was repeated at 72 h for all 6 d time points. Cells were stained with 0.5% crystal violet in 25% methanol, dried, and the stain dissolved in 100 mM sodium citrate in 50% ethanol. Absorbance was measured at 550 nm.

### 2.4. Treatment of Cells and Immunoblotting

Cells were plated in 6-well plates (200,000 cells) and incubated overnight for attachment. One concentration of each phytochemical was used for biomarker analysis. The concentration of phytochemical with the most significant reduction in cell proliferation for that respective cell line was used for treatment of cells for molecular marker analysis. Same vehicle control as described above was used. After 3 d, cells were suspended in radioimmunoprecipitation assay (RIPA) buffer (50 mmol/L Tris-HCl, pH 7.4; 150 mmol/L NaCl; 1% NP40; 0.25% Na-deoxycholate; 1 mM phenylmethylsulfonyl fluoride (PMSF); 1 mM sodium orthovanadate; 1X Roche complete miniprotease inhibitor cocktail). Twenty–forty *μ*g of protein was fractionated using an SDS-PAGE gel, transferred to a nitrocellulose membrane, and blocked with 5% Milk in TBS-T. These membranes were incubated with primary (1 : 1000) followed by an appropriate HRP-conjugated secondary antibodies (1 : 5000) for 1 h at room temperature. Reactive products were visualized by chemiluminescence (Thermoscientific, Rockford, IL) and quantified by densitometry using the Quantity One software (Bio-Rad, Hercules, CA). Membranes were stripped and reprobed for *β*-actin (1 : 1,000) as the loading control. Treatment was replicated independently three times for statistical analysis.

### 2.5. Statistics

The data is represented as mean ± standard error. Cell proliferation data is derived from three to four independent experiments performed in triplicate for each cell line. All treatments were normalized to the vehicle control and fold change was calculated. A *t*-test was used to calculate the differences in mean using Microsoft Excel and a *P* value ≤ 0.05 was considered significant. The relative index (RI) was also calculated using the formula RI = *S*(expected)/*S*(observed) = *S*[*a*] × *S*[*b*]/*S*[*a* + *b*], where *a* and *b* are extracts/compounds and ICI, respectively [28]. An RI = 1 is considered additive, <1 antagonism or absence of synergism, and >1 presence of synergism. 

## 3. Results

### 3.1. Response of Sensitive and Resistant Cell lines to ICI Treatment

 In this study we have used three models of endocrine resistance to test the effect of berry extracts and compounds. The LCC1 cells, originally derived from MCF7 cells, are ER+, E independent, and sensitive to both TAM and ICI. This can also be considered as a model of aromatase inhibitor resistance [6]. LCC9 cells were derived by further culturing LCC1 in sequentially increasing doses of ICI [26]. These cells are ER+, E independent and are TAM and ICI cross-resistant. Previous studies have shown the effect of both RRE and Del in MCF7 cells [22, 29]. We also used ZR75-1 cells, initially derived from a tumor that was unresponsive to TAM [30]. These cells are ER+, PGR+, TAM, and ICI sensitive and express a low level of p53 [31, 32]. ZR75-1R cells were derived from ZR75-1 cells by a similar procedure as for LCC9 cells. They also are cross-resistant to TAM and ICI (data not shown). The BT-474 cells belong to the Luminal B molecular classification along with the ZR75-1 cells [33]. These cells are ER+, PGR+ and overexpress Her 2 [32, 33]. To our knowledge, this is the first study to assess the effects of berry extracts in LCC1, LCC9, ZR75-1, ZR75-1R, and BT-474 cells. This is also the first study to evaluate the effect of berry extracts and phytochemicals on ICI-resistant cell lines. 

 All ICI-sensitive cells were slightly growth inhibited by 1 nM ICI (10–30%). This effect was most prominent after 6 d. In LCC1 cells, 6 d ICI treatment resulted in a 30% decrease in cell proliferation compared to vehicle treatment (0.705; *P* value = 0.0003). In ZR75-1 cells, 1 nM ICI did not cause a significant reduction in cell proliferation at 3 d (fold change = 0.91), but caused a 20% reduction at 6 d (*P* = 0.02). In BT474 cells, ICI treatment (1 nM) alone caused a 13% and 25% (*P* = 0.02) reduction in cell proliferation after 3 and 6 d, respectively. Since the growth inhibitory effect was less than 50% (<IC_50_), we considered 1 nM ICI to be sublethal dose in sensitive cells. On the other hand, resistant cells were not growth inhibited by ICI concentrations of up to 1 *μ*M. Thus, we tested the effects of extracts and compounds to resensitize LCC9 and ZR75-1R at two different concentrations of ICI (1 nM and 1 *μ*M).

### 3.2. Effect of Berry Extracts and Compounds on ICI-Sensitive Cells

#### 3.2.1. Estrogenic Effect in LCC1 Cells

**Figure 1:**
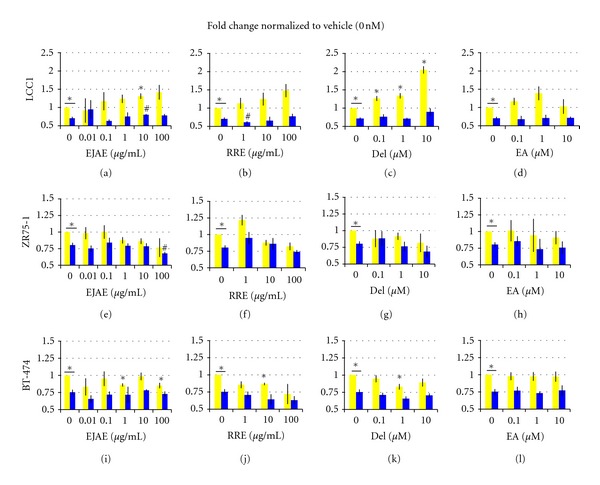
Effect of berry extracts and polyphenols on proliferation of ICI-sensitive (LCC1, ZR75-1, BT-474) cell lines. Cell proliferation results from 6 d treatment of various cell lines with respective berry extract or polyphenol. Yellow (light) bars represent extract/polyphenol only and blue (dark) bars represent extract/polyphenol + 1 nM ICI. Each extract or polyphenol used for treatment was diluted 1000-fold from stock concentration. Vehicle treatment contained 0.01% EtOH and 0.1% DMSO. All treatments were normalized to the appropriate vehicle treatment. Data represented is mean ± standard error of three individual experiments performed in triplicate for each treatment. The means were tested for difference using a *t*-test and a *P* ≤ 0.05 considered significant. The significantly different means compared to vehicle control (*) or ICI-only treatment (^#^) are indicated.

LCC1 cells are ER+ and respond to estrogenic stimuli with increased growth. We found that all berry extracts and compounds had an estrogenic effect on LCC1 cells indicated by the moderate to significant, dose-dependent, increase in cell proliferation after 6 d treatment (Figures 1(a)–1(d)). Further, this effect was found to be ER-mediated since cotreatment of LCC1 cells with ICI abrogated the effects of extracts/compounds (Figures 1(a)–1(d)). These effects were more prominent after 6 d rather than a 3 d treatment. Of note, Del has a significant proliferative effect on LCC1 at 0.1 *μ*M, 1 *μ*M, and 10 *μ*M with a fold change of 1.26 (*P* = 0.046), 1.33 (*P* = 0.035), and 2.04 (*P* = 0.008), respectively (compared to vehicle value of 1.00) (Figure 1(c)). After 6 d treatment, concentrations of 0.1 *μ*M and 1 *μ*M of EA showed a nonsignificant proliferative effect, but 10 *μ*M of EA showed no change in cell proliferation (Figure 1(d)). This shows that there is a specific range of concentrations at which EA may have estrogenic properties.

#### 3.2.2. Growth Inhibitory Effect in ZR75-1 and BT474 Cells

While berry extracts and compounds had significant growth promoting effects on E-independent LCC1 cells, they showed a dose-dependent but moderate growth inhibitory effects of ZR75-1 and BT474 cells grown in E-sufficient medium (Figures 1(e)–1(l)). EJAE alone, dose dependently inhibited the proliferation of ZR75-1 cells after 6 d treatment (Figure 1(e)). This effect was enhanced by cotreatment with ICI but significant only at 100 *μ*g/mL of EJAE (*P* = 0.05; RI = 0.90). In BT474 cells, a variable response was observed with EJAE treatment alone (Figure 1(i)). There were no synergistic or additive effects seen after addition of 1 nM ICI (Figure 1(i)). However, it must be noted that the lower dose had a greater effect when combined with ICI suggesting that doses in physiologically achievable ranges may achieve an additive effect with drug therapy.

A lower dose of RRE (1 *μ*g/mL; 22%) was more effective than the higher dose (100 *μ*g/mL; 13%) in ZR75-1 cells after 3 d. However, at 6 d, only the 100 *μ*g/mL RRE showed a 20% reduction in cell proliferation (Figure 1(f)). This effect was slightly modified to 26% by the addition of 1 nM ICI. In BT474 cells, both 1 and 100 *μ*g/mL RRE showed ≥ 15% reduction in cell proliferation without ICI (*P* ≤ 0.05) after 3 d treatment. However, after 6 d, RRE alone showed a dose-dependent 15–28% reduction (*P* ≤ 0.05 at 10 *μ*g/mL) (Figure 1(j)). The addition of ICI slightly enhanced this effect (Figure 1(j)). However, an RI of 0.86 in ZR75-1 and of 0.89 in BT474 suggests that at higher doses (≥100 *μ*g/mL) RRE and ICI may trend toward antagonizing the action of each other.

In ZR75-1 cells, we observed only 28% and 20% antiproliferative effect at the highest dose of Del (10 *μ*M) at 3 and 6 d, respectively (NS; Figure 1(g)). However, a linear dose response and a synergistic effect were observed when ICI was added (RI ≥ 1.0) (NS; Figure 1(g)). In BT474 cells, after 3 d, the lowest dose of Del was the most effective in curbing proliferation (15% reduction; *P* = 0.02 for 0.1 *μ*M versus ≤5% for 1 and 10 *μ*M). An additive effect was also evident with 1 nM ICI (RI = 1.0; 22% reduction for 0.1 *μ*M Del+ ICI versus ≤13% for ICI alone or other doses of Del+ICI). After 6 d, all doses of Del (0.1 *μ*M–10 *μ*M) achieved *a* ≥ 30% reduction in the presence of ICI showing an RI. = 1.0, suggesting that the effects were additive (Figure 1(k)). EA treatment did not significantly alter the proliferation of either cell line at doses tested with or without ICI after 6 d treatment (Figures 1(h) and 1(m)).

### 3.3. Effect of Berry Extracts and Compounds on ICI-Resistant Cell Lines

#### 3.3.1. Effect of Extracts/Compounds in LCC9 Cells

**Figure 2:**
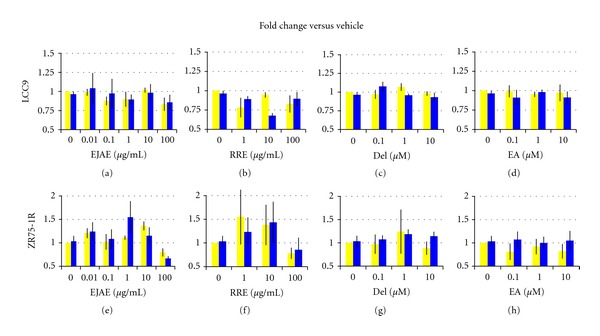
Effect of berry extracts and polyphenols on proliferation of ICI-resistant (LCC9 and ZR75-1R) cell lines. Cell proliferation results from 6 d treatment of various cell lines with respective berry extract or polyphenol. Yellow (light) bars represent extract/polyphenol only and blue (dark) bars represent extract/polyphenol + 1 nM ICI. Each extract or polyphenol used for treatment was diluted 1000-fold from stock concentration. Vehicle treatment contained 0.01% EtOH and 0.1% DMSO. All treatments were normalized to the appropriate vehicle treatment. Data represented is mean ± standard error of three individual experiments performed in triplicate for each treatment. The means were tested for difference using a *t*-test and a *P* ≤ 0.05 considered significant.

In LCC9 cells, EJAE alone (1 *μ*g/mL) caused a modest, but significant, reduction in cell proliferation after 3 d (19%; *P* = 0.028). However this effect was not present at 6 d (Figure 2(a)). EJAE did not significantly resensitize LCC9 cells to either 1 nM (Figure 2(a)) or 1 *μ*M ICI at any concentration tested (data not shown). At 6 d, RRE (10 *μ*g/mL) increased the inhibitory response of LCC9 cells to 1 nM ICI (33%; NS.; RI = 1.34) (Figure 2(b)). However, we did not observe this synergistic effect at 1 *μ*M ICI (data not shown), suggesting that specific cellular responses are elicited at specific doses of the drug (ICI) and the extract (RRE). Del alone had no effect on cell proliferation at 3 or 6 d. In the presence of 1 nM ICI and 0.1 *μ*M Del, there was a 35% induction of cell proliferation (*P* = 0.03) at 3 d. However, this effect was not seen at 6 d (Figure 2(c)). RRE alone (100 *μ*g/mL) caused a 28% and 18% nonsignificant reduction at 3 and 6 d, respectively. This effect was not altered by ICI (1 nM) after 3 d (27%) or 6 d (11%). EA neither had a significant growth inhibiting effect on LCC9 cells after 3 d (data not shown) or 6 d treatment, nor showed an ability to resensitize LCC9 cells to 1 nM ICI (Figure 2(d)).

#### 3.3.2. Effect of Extracts/Compounds in ZR75-1R Cells

**Figure 3:**
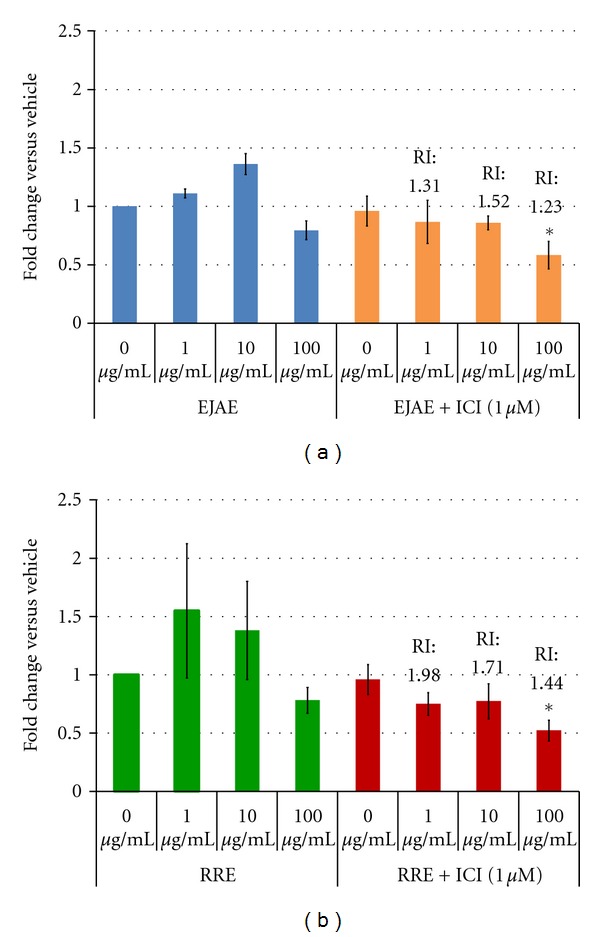
Effect of berry extracts proliferation on ICI-resistant (ZR75-1R) cell line without and with ICI. Cell proliferation results from 6 d treatment of RRE on ZR75-1R. Vehicle treatment contained 0.01% EtOH and 0.1% DMSO. All treatments (including ICI) were normalized to the appropriate vehicle treatment. Data represented is mean ± standard error of three individual experiments performed in triplicate for each treatment. The means were tested for difference using a *t*-test and a *P* ≤ 0.05 considered significant. The significantly different means compared to vehicle control (^#^) or ICI-only treatment (*) are indicated. The relative index (RI) was calculated using the formula RI  = *S*(expected)/*S*(observed) = *S*[*a*] × *S*[*b*]/*S*[*a* + *b*], where *a* and *b*, are extract and ICI, respectively [28]. An RI = 1 was considered additive, <1 antagonism or absence of synergism, and >1 presence of synergism.

ZR75-1R cells were overall more susceptible to the effects of both EJAE and RRE than LCC9 cells. After 3 d, EJAE alone at 100 *μ*g/mL and 0.01 *μ*g/mL showed a reduction in cell proliferation by 20% and 35%, respectively. This suggests a biphasic response with highest and lowest dose eliciting similar effects. However, after 6 d treatment, the reduction was sustained only at the highest dose (Figure 2(e)). In the presence of 1 nM ICI, EJAE (100 *μ*g/mL) showed a reduction in cell proliferation (35%; RI = 1.23). This effect is slightly greater than that seen for EJAE alone (Figure 2(e)). However, in the presence of 1 *μ*M ICI, we observed a linear dose response with 1, 10, and 100 *μ*g/mL of EJAE reducing cell proliferation by 15%, 15%, and 40%, respectively (RI ≥ 1.2 for each treatment) (Figure 3(a)). 

An interesting RRE dose response with an inverted-U curve was observed in ZR75-1R cells. At 6 d, lower doses (1 *μ*g/mL and 10 *μ*g/mL) showed a proliferative effect, whereas higher dose (100 *μ*g/mL) showed a 22% reduction in cell proliferation (Figure 2(f)). Addition of 1 nm ICI does not change this effect (Figure 2(f)). However, in the presence of 1 *μ*M ICI, RRE significantly and dose-dependently inhibited ZR75-1R growth by 25–50% (*P* = 0.05 at 100 *μ*g/mL; RI ≥ 1.4) (Figure 3(b)). Both 1 *μ*g/mL and 10 *μ*g/mL RRE had a highly synergistic effect in the presence of 1 *μ*M ICI with RI values of 1.98 and 1.71, respectively (Figure 3(b)). 

Del treatment of ZR75-1R did not show any significant change in cell proliferation after 3 or 6 d treatment with or without 1 nM ICI (Figure 2(g)). EA treatment of ZR75-1R did not show a change in cell proliferation (Figure 2(h)). However, EA treatment with 1 nM ICI showed a synergistic dose response after 3 d as 1 *μ*M and 10 *μ*M displayed a 15% (RI. = 1.443) and 20% (RI. = 1.555) reduction in cell proliferation, respectively. This shows that EA could resensitize the cells to ICI at 3 d. However, this response was not seen after 6 d EA treatment (Figure 2(h)). 

### 3.4. Effect of Berry Extracts on Estrogen, Progesterone Receptor- and Cell-Death Markers

**Figure 4:**
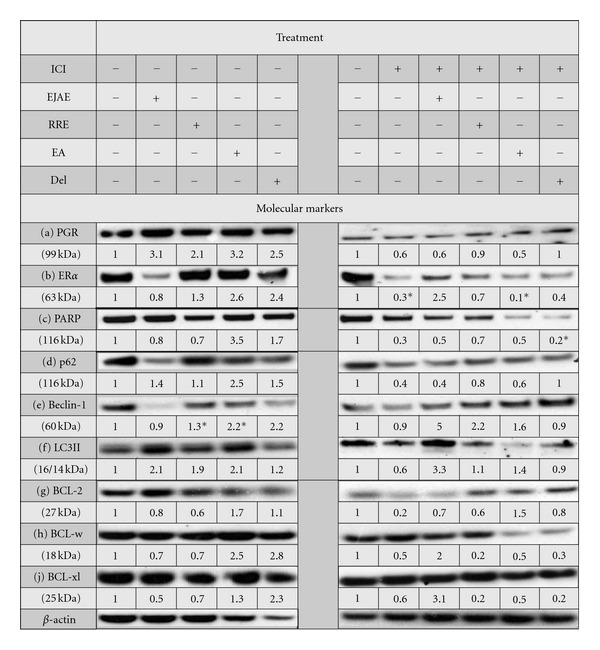
Effect of berry extracts and polyphenols on the expression of nuclear receptors and cell-death markers in ICI-sensitive (LCC1) Cell Lines. Biomarker analysis results from 3 d treatment of berry extracts and polyphenols. Data represented as fold change over the vehicle. Extract only treatment was normalized to nontreated cells (first lane). Extract +ICI was normalized to the appropriate vehicle treatment (first lane). Vehicle treatment contained 0.01% EtOH and 0.1% DMSO. Concentrations of treatments were as follows: ICI = 1 nM; EJAE = 0.1 *μ*g/mL; RRE = 1 *μ*g/mL; EA = 1 *μ*M; DEL = 1 *μ*M. Concentration of extract used showed the most significant reduction in cell proliferation of LCC1 cells after 6 d treatment. Each extract or polyphenol used for treatment was diluted 1000-fold from stock concentration. *designates a significance of *P* ≤ 0.05 compared to vehicle control.

 In order to understand the molecular mechanisms by which berry extracts and its constituents cause the observed effects in LCC1 and LCC9 cells, we assessed the levels of various molecular markers in these cells after treatment with berry extract/compound alone or in the presence of ICI. Since the antiproliferative effects of the compounds were evident at 3 d, we chose this time point to study molecular mechanisms. Also, we specifically chose those concentrations of extracts/compounds that produced the greatest reduction in cell proliferation at 6 d (Figures 1(a)–1(h)). As expected in LCC1 cells, EJAE and Del alone showed an estrogenic response with an observed increase of 3- and 2.5-fold, respectively, in progesterone receptor (PGR) expression (Figure 4(a)). RRE and EA also increase PGR expression by greater than 2-fold. This increase was completely reversed by cotreatment with ICI (Figure 4(a)). Interestingly, EA and Del alone also raised ER*α* levels (Figure 4(b)). We also assessed molecular markers of cell death (PARP cleavage), autophagy (p62, Beclin 1, light chain-3 (LC3)), and the levels of the antiapoptotic B-cell lymphoma (BCL) family members (BCL2, BCLw, and BCLxl). It has been previously shown that combined knockdown of BCL2 and BCLw leads to resensitization of LCC9 cells to TAM and ICI [34]. BCL2 also interacts with Beclin 1 to inhibit autophagy [35]. 

Although we observed a clear increase in PARP expression by EA alone and a reduction in levels after ICI treatment across all extracts, we did not see cleavage of PARP in any of the treatments (Figure 4(c)). EJAE, RRE, and Del caused a 2-fold induction in LC3II levels, which was reversed by the addition of ICI, except for EJAE (Figure 4(f)). EJAE + ICI caused a 3.3-fold induction of LC3II in LCC1 cells (Figure 4(f)). On the other hand, EJAE, Del, and EA increased the levels of p62 to various extents (1.4 to 2.5-fold; Figure 4(d)). As expected, ICI treatment reduced p62 levels by 60%, suggesting active autophagy. EJAE did not alter this response; however, RRE, EA, and DEL all reversed this reduction. RRE and EA treatments significantly increased the levels on Beclin-1 (Figure 4(e)). EJAE induced Beclin-1 levels by 5-fold in the presence of ICI (Figure 4(e)). The effect of extracts/compounds on the BCL2-family members was neither uniform nor significant (Figures 4(g)–4(j)). EA increased the expression of BCL-2 > 1.5-fold without or with ICI (Figure 4(g)). BCLw and BCLxL expression was induced by EA and Del alone and EJAE + ICI (Figures 4(h) and 4(j)). 

**Figure 5:**
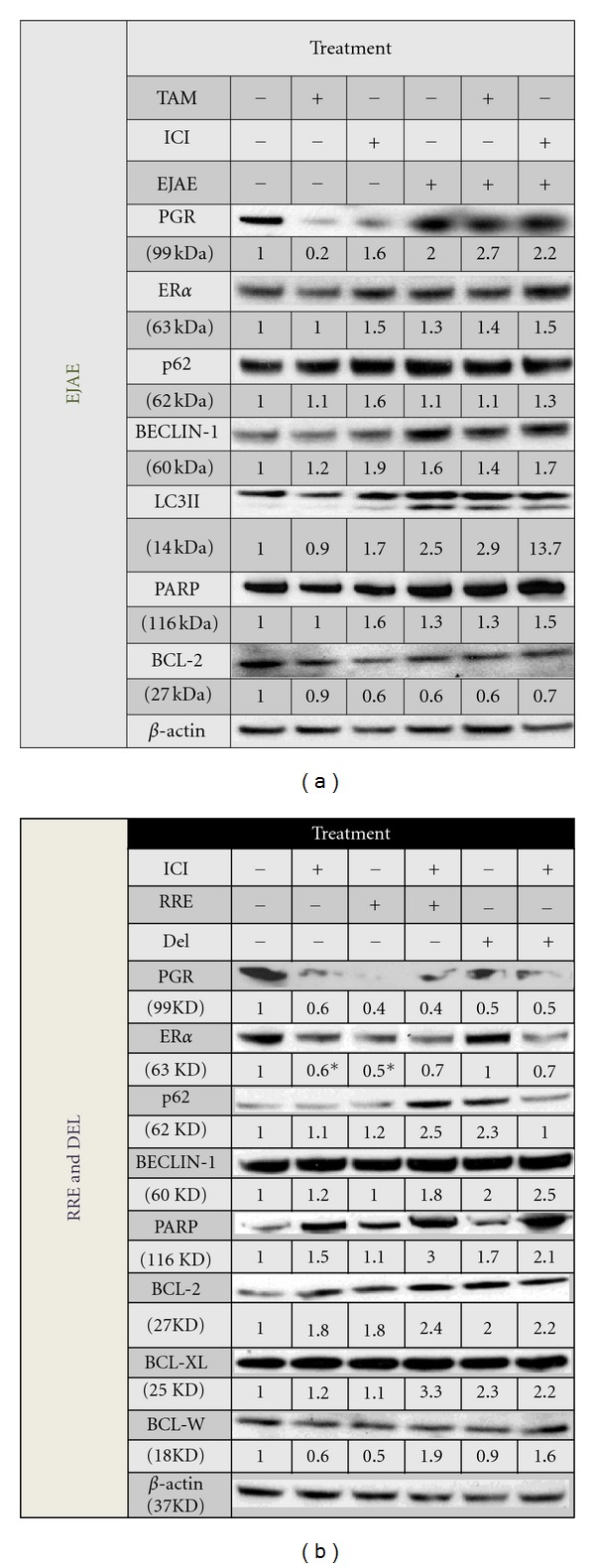
(a) and (b) Effect of EJAE and RRE on the expression of nuclear receptors and cell-death markers in ICI-resistant (LCC9) cell lines. Biomarker analysis results after 3 d treatment of EJAE with and without drug treatments. Data represented as fold change over the vehicle. All treatments were normalized to vehicle treatment (first lane). Vehicle treatment contained 0.01% EtOH and 0.1% DMSO. Concentrations of treatments were as follows: TAM (4-0HT) = 100 nM; ICI = 1 nM; EJAE = 100 *μ*g/mL; RRE = 10 *μ*g/mL; and DEL = 1 *μ*M. Concentration of extract used showed the most significant reduction in cell proliferation of LCC9 cells after 6 d treatment. Each extract or polyphenol used for treatment was diluted 1000-fold from stock concentration. *designates a significance of *P* ≤ 0.05 compared to vehicle control.

In LCC9 cells, EJAE induced PGR levels without altering ER*α* levels (Figure 5(a)). EJAE also induced Beclin-1 levels, both in the presence and absence of ICI. EJAE + ICI treatment increased LC3II levels by 13.7-fold over vehicle or ICI-only treatment, suggesting that EJAE may increase autophagosome formation in LCC9 cells. However, we failed to see a baseline increase in LC3II with ICI-only treatment. We did not observe PARP cleavage or a significant change in BCL2 levels. On the other hand, RRE + ICI, Del alone, and Del + ICI showed an across-the-board increase in the expression of all BCL2-family proteins (BCL2, BCLw, and BCLxl), as well as Beclin-1. RRE + ICI treatment and Del increased p62 expression 2–2.5 fold suggesting an inhibition of autophagy.

## 4. Discussion

In this study, we tested whether berry extracts/compounds synergize with a sublethal dose of ICI and increase drug response in ICI-sensitive cell lines. We also tested whether cotreatment of berry extracts reverses the resistant phenotype in ICI-resistant cells, thereby leading to an increased cell death in the presence of ICI. 

We used multiple cell lines with different molecular characteristics that are representative of the ER+ tumors commonly seen in the patient population. The ICI-sensitive cells used can be divided into Luminal A (ER+, PGR+, HER2-; LCC1) and B (ER+, PGR+, HER2+; ZR75-1 and BT-474) subtypes [33] and the resistant cells derived from the same subtypes (LCC9 from LCC1 and ZR75-1R from ZR75-1). Further, these cells were also cultured in different media with LCC1 and LCC9 in E-deficient medium (<10^−12^ M E) and all other cell lines in an E-sufficient medium (~10^−8 ^M E). In addition to this, we tested two berry extracts (EJAE and RRE) and their representative compounds (Del and EA). This complex design was purposely selected to mimic the complex nature of breast cancer as it presents in the clinic. In the clinic, we are likely to see patients, both pre- (E sufficient) and postmenopausal (E deficient), with ER+ tumors of either subtypes (Luminal A and B) that will be treated with ICI. Furthermore, dietary recommendations will involve whole fruits and not pure components. So we chose whole berry extracts from two different varieties of berries. Finally, we included individual components from each berry extract to determine the contribution of the food matrix. 

 The results from the cell proliferation studies are straightforward and answer many of these questions. Both berry extracts and compounds synergize with a sublethal dose of ICI (1 nM) to cause an inhibition of cell proliferation in ICI-sensitive cell lines. The berry extracts/compounds are most effective in BT-474 cells and least effective in LCC1 cells. The effects of the extract/compounds alone vary greatly with the doses used and the type of cell-line. We observed that both extracts and compounds had an estrogenic effect on LCC1 cells grown in an E-deficient medium (Figures 1(a)–1(d)). This effect was significant and dose dependent for EJAE and Del (Figures 1(a) and 1(c)). However, the same extracts/compounds showed a dose-dependent inhibition of cell proliferation in ZR75-1 and BT-474 cells grown in E-sufficient medium (Figures 1(e)–1(l)). This effect is similar to that observed with TAM, which stimulates MCF-7 cell growth under E-deficient, and inhibits proliferation under E-sufficient conditions (Aiyer, unpublished results). The SERM activity of TAM reported in literature also suggests such effects [6, 36]. Many polyphenolic compounds including those present in berries have been reported to show SERM-like effects [3]. Further, they also show a dose-dependent selective recruitment of coactivators and repressors to the ER, which dictates whether these compounds will act as estrogens or antiestrogens [3, 37]. There are indications toward such effects in our study. For example, RRE shows a slight estrogenic effect (increased cell growth) at 1 *μ*g/mL, whereas inhibits growth at 10 and 100 *μ*g/mL (Figure 1(g)). Regardless of their effects alone, all extracts/compounds tested show varying degrees of either additive or synergistic response with ICI in sensitive cells. 

 Another important difference between the cell lines selected is the HER2 status. Both BT-474 and ZR-75 cells are HER2+, whereas LCC1 cells are not [6, 33]. Although all cell lines possess ER, the constitutive levels of this receptor vary greatly among these cell lines at baseline (Aiyer, unpublished results). Thus, the differential effect of berry extracts/compounds seen among various cell lines could be partially explained by these differences. Many berry compounds have been shown to deactivate tyrosine kinase signaling and can inhibit HER2-mediated effects on cell proliferation (reviewed in [3]). It is clear that in LCC1 cells, the proliferative responses seen are ER*α*-mediated. There is an extract-induced increase in PGR levels (Figure 4(a)), which suggests a classic genomic response due to ER activation [38]. Additionally, treatment with extracts seems to stabilize ER*α* levels and antagonize the ICI-mediated degradation of ER*α* in LCC1 cells (Figure 4(b)). The implications of such effects on long-term ICI-treatment and resistance development must be explored further.

 In ICI-resistant cells, neither extracts nor pure compounds resensitized the cells to lower dose of ICI (1 nM) (Figures 2(a)–2(h)). The resistant cells continue to grow in the presence of ICI up to 1 *μ*M and hence may be resistant to many chemopreventive agents tested at lower doses. However, both EJAE and RRE at all concentrations tested synergize with 1 *μ*M ICI to cause a significant reduction in resistant (ZR75-1R) cell growth (Figure 3). A limitation of this study is the lack of clear mechanistic detail regarding how berry extracts and compounds reduce cell growth. We studied the induction of autophagy using p62, LC3II, and Beclin 1 as surrogate markers. Many compounds present in berries have been shown to induce autophagy-associated cell death [3, 39–41]. However, in terms of endocrine resistance, autophagy is possibly a survival mechanism and inhibition of autophagy alone, or in combination with BCL2+BCLw coinhibition, resensitizes the resistant cells to AEs [34]. There is some evidence to suggest that RRE, Del, and EA may interfere with autophagy in LCC1 cells. They reverse the ICI-induced degradation of p62 to various extent (Figure 4(d)). However, the changes seen in LC3II and Beclin 1 levels contradict this argument. It is clear that EJAE ± ICI consistently increases autophagy in both LCC1 and LCC9 cells. Since we have not directly counted LC3 punctae formation, the data is inconclusive as to whether berry extracts/compounds inhibit autophagy and the exact mechanisms by which they do so. However, we only analyzed these markers in LCC1 and LCC9 cells, where the effects of the extracts/compounds were minimal. The assessment of the markers in other cell lines is currently underway and should provide a clearer understanding of the various mechanisms by which berry extracts/compounds increase cell-line responsiveness to ICI.

 Other research into berry extracts and compounds have shown similar results, although specific mechanisms vary. Previous studies show that the IC_50_ of RRE in MCF7 cells grown in E-sufficient medium is 190 *μ*g/mL at 48 h [22]. This is comparable to the effect seen in the ZR75-1 and BT474 cells, grown under similar conditions, in this study. EJAE has been tested in breast cancer cell lines such as MCF7-aro and MDA-MB-231 with an IC_50_ of 27 and 40 *μ*g/mL, respectively, at 72 h [24]. Aqil et al. [25] have shown that hydrolyzed extract of the Jamun fruit pulp was effective in reducing the growth of the non-small cell lung cancer cell-line A549 with an IC_50_ of 59 *μ*g/mL at 72 h. Such studies have typically reported the cytotoxic effects of extracts/compounds within 72 h. By contrast, our data presents the effect at both 72 h (3 d) and at 6 d. It is seen that many of the effects at 3 d are not carried over at 6 d. This could be due to the clonogenic expansion of the cells that were initially unresponsive to the chemopreventive agent. Since effects of dietary bioactive compounds on cancer cells are a chronic process, 2 or 3 d cell proliferation studies, as is typically performed for a majority of these agents, may lead to the overrepresentation of the effectiveness of such compounds. 

 In conclusion, using a clonogenic assay, we have shown that berry extracts and compounds can increase the cell-death response of ICI-sensitive breast cancer lines to a sublethal dose of ICI (1 nM, <IC_50_ dose). Further, we also show that this response is largely additive rather than synergistic. Additionally, berry extracts resensitize ICI-resistant cells to ICI treatment showing a synergistic response, especially at the higher dose (1 *μ*M). Finally, we found that extracts were more effective at lower, physiologically relevant doses than at higher experimental doses in some cell lines. These results indicate that berry extracts and compounds can potentially interact with ICI in breast epithelial cells to alter drug-response. Further *in vivo* research is warranted regarding the implications of such food-drug interactions in response to ICI treatment and the development of drug resistance.
